# Publisher’s Note

**DOI:** 10.1080/21655979.2024.2326368

**Published:** 2024-03-01

Due to a publisher error, a retraction notice was incorrectly issued on the article ‘MicroRNA-4521 targets hepatoma up-regulated protein (HURP) to inhibit the malignant progression of breast cancer’, https://doi.org/10.1080/21655979.2021.1996016. We have now removed the retraction notice.

We apologise to the authors for this error.

